# Mechanisms of palmitate-induced cell death in human osteoblasts

**DOI:** 10.1242/bio.20136700

**Published:** 2013-11-06

**Authors:** Krishanthi Gunaratnam, Christopher Vidal, Ross Boadle, Chris Thekkedam, Gustavo Duque

**Affiliations:** 1 Ageing Bone Research Program, Sydney Medical School Nepean, The University of Sydney, Penrith, NSW 2750, Australia; 2 Electron Microscope Laboratory, ICPMR and Westmead Research Hub, Westmead, NSW 2145, Australia

**Keywords:** Fatty acids, Apoptosis, Autophagy, Osteoporosis, Osteoblasts

## Abstract

Lipotoxicity is an overload of lipids in non-adipose tissues that affects function and induces cell death. Lipotoxicity has been demonstrated in bone cells *in vitro* using osteoblasts and adipocytes in coculture. In this condition, lipotoxicity was induced by high levels of saturated fatty acids (mostly palmitate) secreted by cultured adipocytes acting in a paracrine manner. In the present study, we aimed to identify the underlying mechanisms of lipotoxicity in human osteoblasts. Palmitate induced autophagy in cultured osteoblasts, which was preceded by the activation of autophagosomes that surround palmitate droplets. Palmitate also induced apoptosis though the activation of the Fas/Jun kinase (JNK) apoptotic pathway. In addition, osteoblasts could be protected from lipotoxicity by inhibiting autophagy with the phosphoinositide kinase inhibitor 3-methyladenine or by inhibiting apoptosis with the JNK inhibitor SP600125. In summary, we have identified two major molecular mechanisms of lipotoxicity in osteoblasts and in doing so we have identified a new potential therapeutic approach to prevent osteoblast dysfunction and death, which are common features of age-related bone loss and osteoporosis.

## Introduction

Osteoporosis is a major public health issue in the older population that is characterized by low bone mass, deterioration of bone microarchitecture, and increased susceptibility to fractures (Pei and Tontonoz, 2004). During the natural process of aging, bone mass declines while marrow fat increases (Gimble et al., 2006; Rosen and Bouxsein, 2006). In recent years, there has been increasing focus on the functional relationship between fat and bone (Duque, 2008; Rosen and Bouxsein, 2006) based on the idea that decreasing fat infiltration of bone may lead to an increase in bone mass (Pei and Tontonoz, 2004; Rosen and Bouxsein, 2006), which would contribute to fracture prevention.

Two major therapeutic approaches to osteoporosis that target the relationship between fat and bone have been proposed based on the fact that fat and bone share not only the same precursor cell, known as the mesenchymal stem cell (MSC), but also the same microenvironment (bone marrow milieu) (Rosen and Bouxsein, 2006). The first approach is based on the potential for inhibition of adipogenesis to facilitate osteoblastogenesis and bone formation (Duque et al., 2013). The second approach is based on recent *in vitro* evidence for the toxic effect that mature adipocytes can have on bone, including cell dysfunction and cell death (Elbaz et al., 2010). This toxic effect is known as lipotoxicity. The approach aims to protect osteoblasts from lipotoxicity by preventing adipocytes from secreting lipotoxic factors such as fatty acids (FA) and adipokines.

Lipotoxic cell dysfunction and apoptotic cell death induced by adipocyte-secreted factors (known as lipoapoptosis) result from an overload of lipids within an organ or tissue (Martino et al., 2012; Unger and Orci, 2002; Shimabukuro et al., 1998). This lipid overload, which is particularly significant in the pathology of obesity, type 2 diabetes and heart failure (Lee et al., 1994; Unger and Orci, 2002; Shimabukuro et al., 1998) involves cell dysfunction, autophagy, and activation of lipoapoptosis. The mechanisms of lipoapoptosis in organs such as heart, liver, and pancreas have been extensively studied (Borradaile and Schaffer, 2005; Cazanave et al., 2011; Unger and Zhou, 2001). In bone, two *in vitro* studies reported the presence of lipotoxicity after osteoblasts were exposed to adipocyte-secreted factors (Elbaz et al., 2010; Kim et al., 2008). Among these adipocyte-secreted factors, FA (predominantly palmitic acid [PA]) were identified as toxic to osteoblasts; however, the intrinsic mechanisms for this lipotoxicity remain unknown. Given that autophagy and apoptosis are the most common mechanisms of lipotoxicity in other organs and tissues (Martino et al., 2012; Unger and Orci, 2002), and that apoptosis is a common feature in age-related bone loss and osteoporosis (Manolagas, 2000), we focused our analysis on identifying intrinsic mechanisms of autophagy and apoptosis in human osteoblasts (Ob) exposed to PA *in vitro*. To offer a potential therapeutic approach to lipotoxicity in bone, we also tested whether inhibition of PA-induced autophagy and apoptosis is feasible and has a beneficial effect on osteoblast survival.

## Results

### PA induces cell death in Ob in a dose-dependent manner

**Fig. 1.**
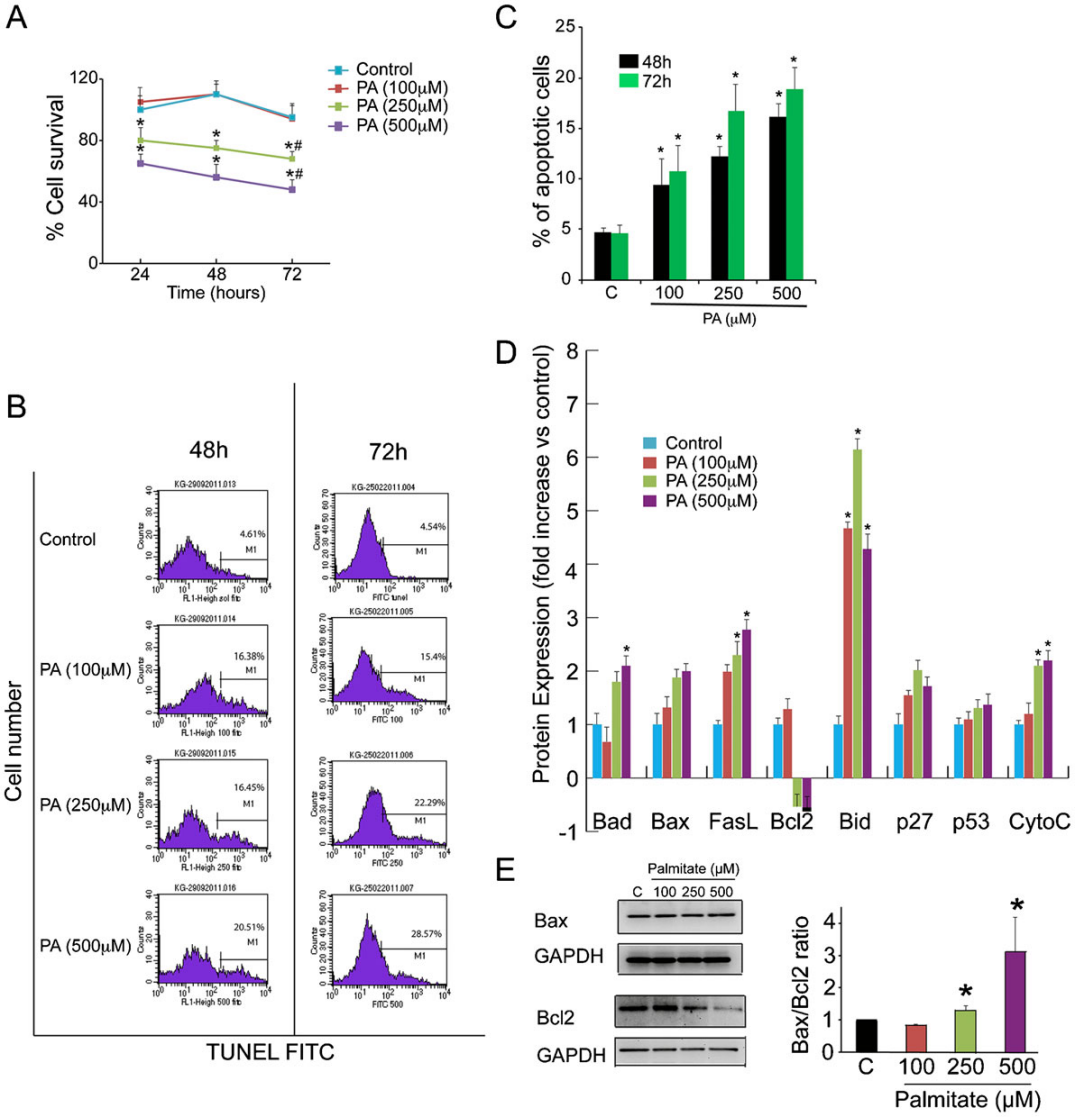
PA induces cell death in human osteoblasts in a dose-dependent manner. (A) Human osteoblasts (Ob) were treated with PA (100, 250, and 500 µM) for 24, 48 and 72 h for cell survival assay (MTS). Data are mean percentage of three independent assays. The percentage absorbance of treated Ob was expressed relative to absorbance of control Ob, which was set at 100%. *P<0.001, vs. vehicle-treated control; #*P*<0.05 vs. % at 24 h for each condition. (B and C) Ob were treated with PA (100, 250 and 500 µM) for 48 and 72 h. Ob were analysed for the relative percentage of apoptotic cells was measured by TUNEL. The data are representative of three different experiments and are shown as mean±SD. *P<0.05 vs. vehicle-treated control. (D) Proteomic analysis of apoptotic pathways in Ob treated with either PA (100, 250 and 500 µM) or vehicle (control) for 48 h. Results were reported as mean ± SEM for at least three analyses for each sample. (E) Western blot of Ob treated with PA (100, 250, and 500 µM) demonstrating Bax and Bcl2 expression at 48 h. The right hand panels show quantification of the Bax/Bcl2 ratio against GAPDH using image J software. *P<0.05 vs. vehicle-treated control.

To examine the effect of PA on Ob survival, we performed an MTS assay in PA- and vehicle-treated cells. This homogeneous colorimetric assay determines the number of viable cells in either PA-treated or untreated conditions. Ob survival was significantly decreased in Ob in the presence of PA (250 and 500 µM) at different time points (24, 48, 72 h) (*P*<0.001) (Fig. 1A). Treatment with 250 µM PA resulted in ∼13%, 20% and 20% reduction of survival at 24, 48 and 72 h respectively. Treatment with 500 µM PA resulted in ∼22%, 33% and 40% reduction of survival at 24, 48 and 72 h respectively. In contrast, treatment with a lower dose of PA (100 µM) had no significant effect on cell survival compared with the vehicle-treated control.

We then measured apoptosis in PA-treated Ob using flow cytometry of TUNEL assay (Fig. 1B,C). The percentage of TUNEL-positive apoptotic cells at 48 h was significantly higher in PA-treated Ob (∼12%, 16% and 20% in cells treated with 100, 250 and 500 µM PA respectively) than vehicle-treated cells (5.6%) (*P*<0.05) and a similar increase in apoptosis was seen at 72 h (Fig. 1B,C).

### Apoptosis pathways activated by PA in Ob

We have previously reported that PA induces apoptosis in osteoblasts *in vitro* (Elbaz et al., 2010). However, the PA-activated apoptotic pathways in human Ob remain unknown. There are two main pathways involved in apoptosis: the extrinsic pathway, which requires transmembrane death receptor-mediated interactions, and the intrinsic pathway, which initiates apoptosis by mitochondria-mediated stimuli. To identify the apoptotic pathway(s) activated by PA, we performed a comprehensive proteomic analysis using a protein array with antibodies against 43 apoptosis-related proteins. The results indicated that PA activated elements of both pathways in Ob. We found significantly increased expression of Fas ligand (FasL), a major component of the extrinsic pathway, in PA-treated Ob. In addition, PA increased the expression levels of the pro-apoptotic mitochondrial proteins Bad, Bid and Cytochrome C concomitant with a decreased expression of the anti-apoptotic protein Bcl2 (Fig. 1D). We also found a non-significant increase in the expression of the apoptotic proteins Bax, p27 and p53 in palmitate-treated Ob (Fig. 1D). Subsequently, to determine whether the balance between the pro-apoptotic Bax and anti-apoptotic factor Bcl2 was affected by the presence of PA, we performed western blot analysis and calculated the Bax/Bcl2 expression ratio. As shown in Fig. 1E, treatment with PA resulted in a significant decrease in Bcl2, stable levels of Bax expression and a significant increase in the Bax/Bcl2 ratio (*P*<0.05), which indicate cell susceptibility to apoptosis.

### Inhibition of lipoapoptosis in Ob by inhibition of the JNK mitochondrial pathway

**Fig. 2.**
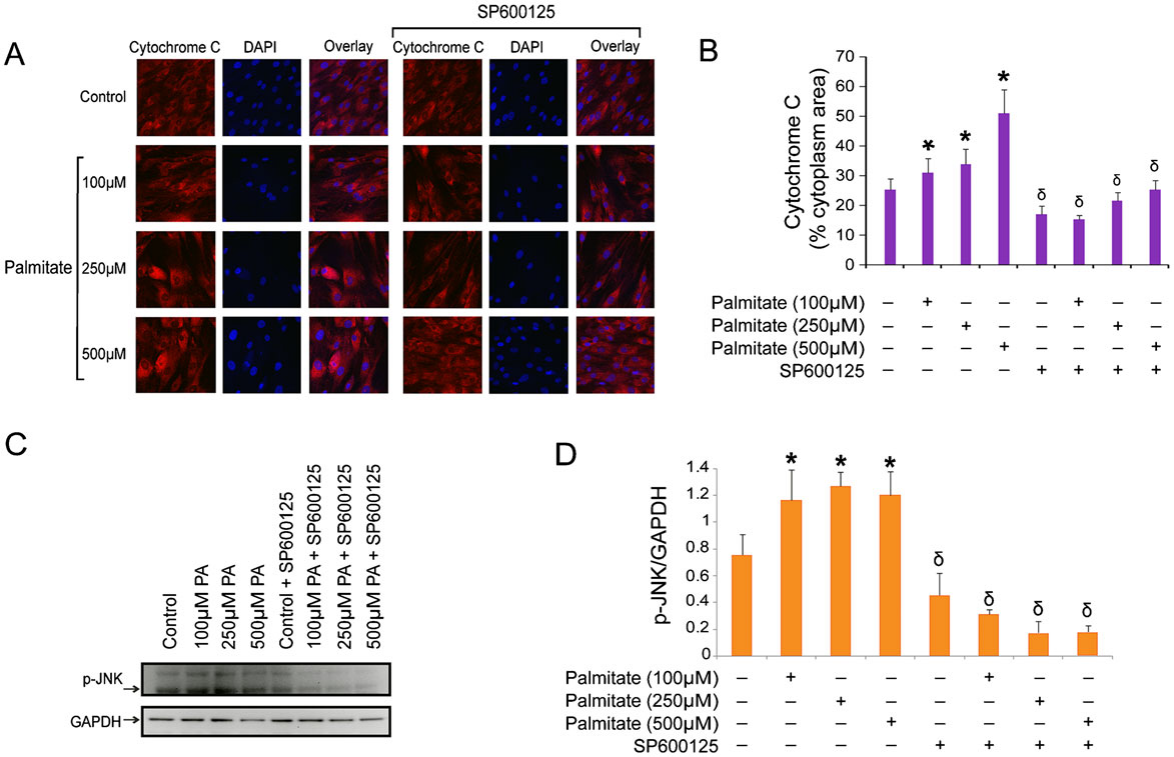
Inhibition of JNK phosphorylation prevents PA-induced apoptosis in Ob. (A) Ob were grown on glass coverslips, treated with PA in the presence or absence of SP600125 for 48 h, and processed for immunofluorescence. Cells were fixed in 4% paraformaldehyde, permeabilized and stained with anti-mouse cytosolic cytochrome C followed by anti-mouse Alexa555. All images are at equal magnification and were processed and acquired identically (60×). (B) Fluorescence for cytochrome C was quantified using Image J software in 10 cells per field in 10 fields from two independent coverslips. The numbers represent the % of cytosol area occupied by red fluorescence (cytochrome C). *P<0.01 PA-treated vs. vehicle-treated control. δ*P*<0.001 SP600125-treated vs. their corresponding untreated cell group according to the dose of PA. (C) Western blots analysis of cells treated with PA (100, 250 and 500 µM) for 48 h, with or without SP600125 for p-JNK and GAPDH. (D) Quantification of p-JNK expression against GAPDH using image J software. *P<0.05 PA-treated vs. control. δ*P*<0.01 SP600125-treated vs. their corresponding untreated cell group according to the dose of PA.

Mitochondrial activation of apoptosis is naturally followed by activation of the Jun kinase (JNK) apoptotic pathway. Activation of the JNK apoptotic pathway induces activation of Bak and Bax, permeabilization of the outer mitochondrial membrane, and release of cytochrome C into de cytosol, which finally activates apoptosis. Therefore, we tested whether using an inhibitor of JNK phosphorylation would have an anti-apoptotic effect on our model. As shown in Fig. 2A,B, and in agreement with our array results, expression of cytosolic cytochrome C was increased in Ob treated with the higher doses of PA. This effect was significantly decreased after treating the cells with SP600125, a potent inhibitor of JNK phosphorylation (*P*<0.001). In addition, PA stimulated JNK phosphorylation in a dose-dependent manner (Fig. 2C), and Ob treated with SP600125 showed a reduction in phosphorylated JNK (Fig. 2C,D), which is consistent with a protection against PA-induced apoptosis by releasing lower levels of cytochrome C into the cytosol (Fig. 2A).

### PA activates autophagy and nuclear fragmentation in Ob

**Fig. 3.**
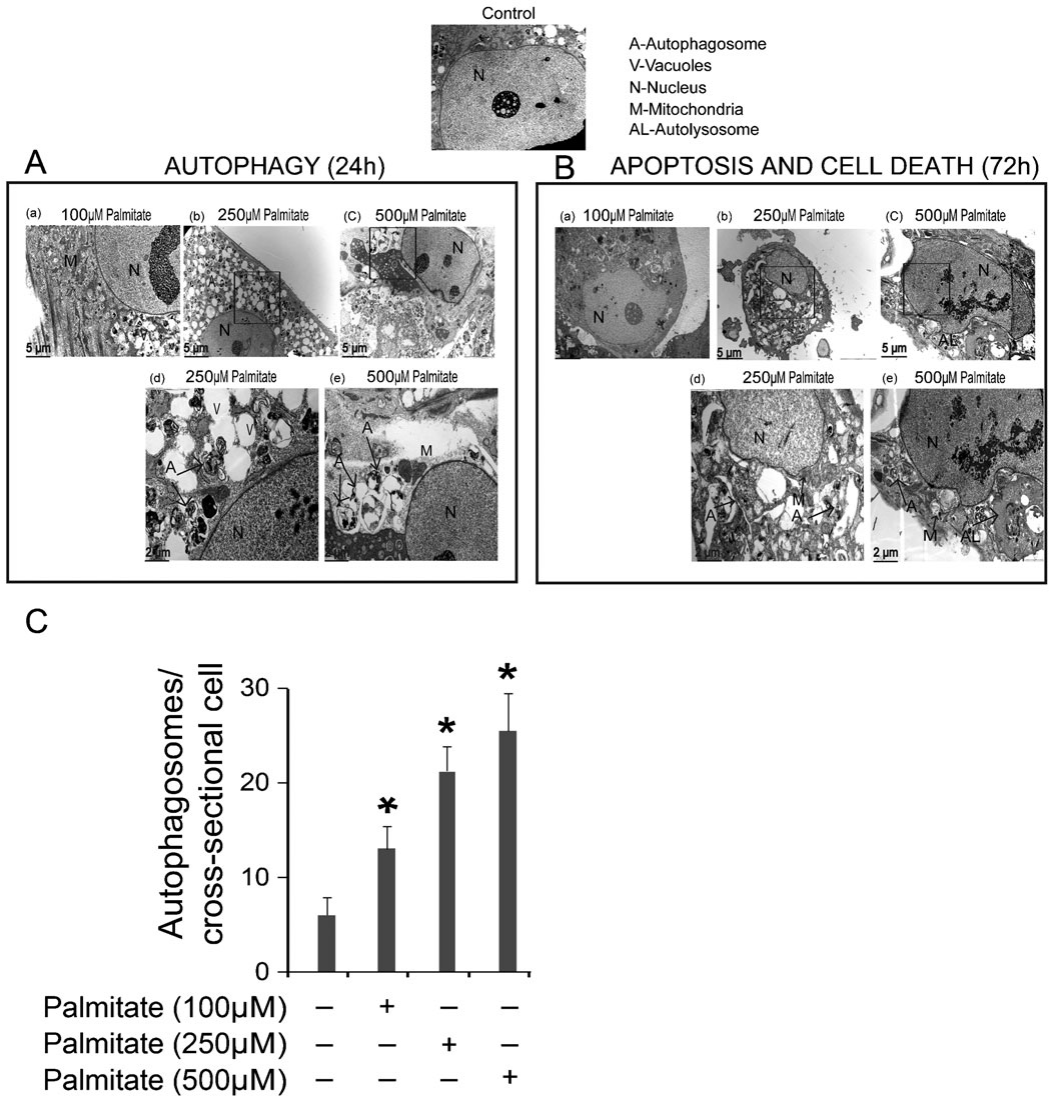
Ultrastructure of Ob treated with PA. (A) Transmission electron micrograph of Ob showing autophagic vacuoles and autophagosomes after 24 h treatment with PA. The cytoplasm and nucleus of untreated cells appear normal. Panels (a–c) show cells treated with increasing doses of PA (100, 250, and 500 µM). Cells exhibited not only cytoplasmic lipid droplets but also the characteristic ultrastructural morphology of autophagy: vacuoles, isolated double-membrane and double-membrane autophagosomes, which engulfed the cytoplasm fraction and organelles, were distributed throughout the cytoplasm. Panels (d–e) are higher magnification images and show examples of autophagosomes. (B) The initial autophagy changes were followed by cell shrinkage and nuclear fragmentation after 72 h of treatment with PA. Panels (a–c) show cells treated with increasing doses of PA (100, 250, and 500 µM). Morphological features of apoptosis and autophagy coexisted in Ob treated at the higher doses of PA. Panels (d–e) are higher magnification images showing typical autolysosomes, mitochondrial deformity and nuclear fragmentation. (C) Quantification of autophagosomes in Ob treated with PA for 24 h. *P<0.01 vs. control.

PA is known to activate autophagy in several cell types including beta cells (Martino et al., 2012; Choi et al., 2009) and endothelial cells (Kabeya et al., 2000). Autophagy involves maturation of autophagosomes, followed by fusion of autophagosomes and lysosomes (autolysosomes) and degradation of damaged organelles or unwanted cellular components. Activation of autophagy could be synchronous with, or could precede, the activation of apoptosis. We measured activation of autophagy by identifying the formation of autophagosomes using electron microscopy (EM) (Fig. 3A–C). More autophagosomes were detected in Ob treated with PA than in vehicle-treated cells (Fig. 3A,C) (*P*<0.01). In addition, PA predominantly induced autophagosomes in Ob within the first 24 h (Fig. 3A,C), following by induction of autolysosomes and nuclear fragmentation at a later stage (72 h) (Fig. 3B).

**Fig. 4.**
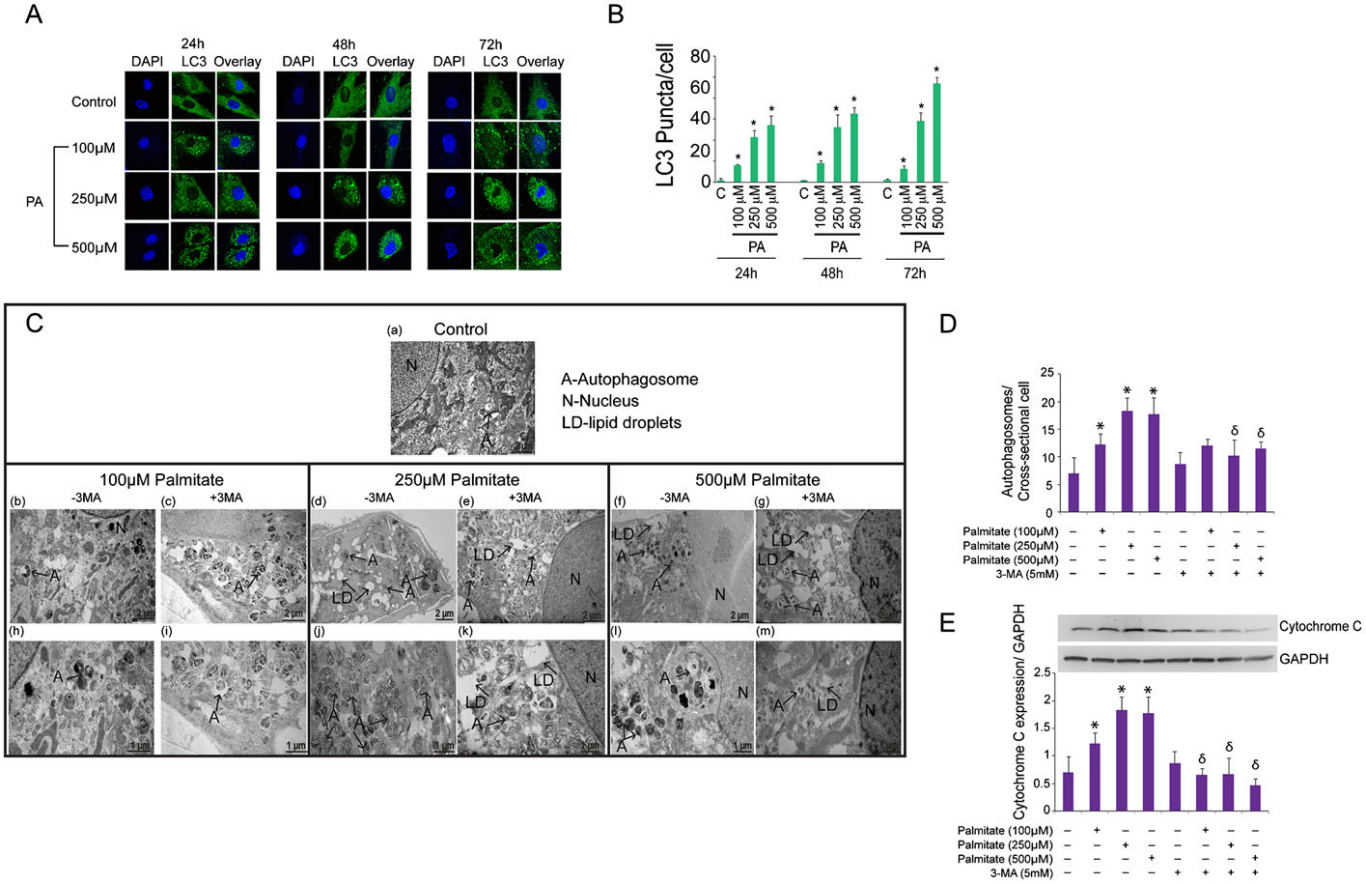
PA induces autophagy in Ob, which is prevented by 3MA. (A) Ob were grown on glass coverslips, treated with PA (100, 250, and 500 µM) for 24, 48 and 72 h and processed for immunofluorescence. Whole cells were fixed in 4% paraformaldehyde, permeabilized and stained with rabbit anti-LC3 followed by anti-rabbit Alexa 488. All images are at equal magnification and were processed and acquired identically (60×). (B) Quantification of the number of LC3 puncta per cell in ten fields from ten different cells in two independent coverslips after treatment with PA for 24, 48 and 72 h. *P<0.01 vs. control. (C) Changes in autophagy induced by 3MA treatment in PA-treated Ob. Ob were treated with PA 100 µM PA (b,h), 250 µM PA(d,j), or 500 µM PA(f,l) for 48 h and processed for EM. Higher number and larger autophagosomes were seen at higher concentrations of PA. Other groups of Ob were treated with 3MA (5 mM) together with 100 µM PA (c,i), 250 µM PA (e,k), or 500 µM PA (g,m) for 48 h. The larger autophagosomes were observed at higher concentrations of PA (j,l). A, autophagosomes; N, nucleus; LD-lipid droplets. (D) Quantification of autophagosomes in Ob treated with PA (100, 250, and 500 µM) with or without 3MA for 24 h. *P<0.01 vs. control. δ*P*<0.01 3MA-treated vs. their corresponding untreated group according to the dose of PA. (E) Quantification of cytochrome C expression against GAPDH using image J software. *P<0.05 vs. control. δ*P*<0.01 3MA-treated vs. their corresponding untreated group according to the dose of PA.

To confirm the presence and extension of PA-induced autophagy in Ob, we assessed the presence of the lipidated form of microtubule-associated protein 1 light chain 3 (LC3) known as LC3II. Lipidation of LC3I into LC3II occurs in the presence of phospholipids and FA in the cytoplasm. LC3II is then a useful marker of autophagic membranes, as it migrates to an apparently lower *M*r position (LC3II) by electrophoresis, and autophagosomes are visualized as bright GFP-LC3 puncta (Kabeya et al., 2000; Tanida et al., 2004). Autophagy was assessed by quantification of LC3 puncta after treatment with PA for 24, 48 and 72 h. Higher number of LC3 puncta/cell was observed following exposure to higher concentrations of PA (Fig. 4A,B) (*P*<0.001).

### 3MA inhibits autophagy and prevents apoptosis in PA-treated Ob

3-methyladenine (3MA) is a potent inhibitor of PI3-kinase (PI3K) that inhibits autophagy (Petiot et al., 2000; Seglen and Gordon, 1982). To evaluate whether 3MA inhibits autophagic activity in our model, we treated Ob with PA in the presence or absence of 5 mM 3MA, which has been reported to have a strong inhibitory effect on autophagy in other cell models (Choi et al., 2009). As shown in Fig. 4C,D, Ob treated with 3MA showed a reduction in the number of autophagosomes quantified by EM at 72 h (*P*<0.05).

Finally, we investigated the effect of 3MA on expression of cytochrome C in Ob treated with PA (Fig. 4E). 3MA treatment decreased levels of cytochrome C expression in PA-treated Ob (Fig. 4E). These findings correlated with lower levels of nuclear fragmentation found in the EM images obtained from 3MA-treated cells (Fig. 4C).

## Discussion

Both autophagy and apoptosis have been identified as key determinants in skeletal maintenance (Hocking et al., 2012; Manolagas and Parfitt, 2010). It has been proposed that during the normal aging process in bone there is an increase in apoptosis and autophagy failure, and that both these processes are increased still further in osteoporotic bone (Manolagas and Parfitt, 2010). However, the mechanisms that induce autophagy and apoptosis in bone cells remain unclear.

Another common feature in aging bone is the increasing presence of fat within the bone marrow milieu (Meunier et al., 1971). High levels of marrow fat secrete FA and adipokines that have been associated with lipotoxicity in bone cells (Elbaz et al., 2010) and in other organs (Unger and Zhou, 2001). Recently, in a study looking at changes in marrow fat composition in diabetic and non-diabetic post-menopausal women, Patsch et al. (Patsch et al., 2013) reported that more importantly that the levels of fat infiltration within the bone marrow of human vertebrae, levels of saturated fat (measured by magnetic resonance) are a risk factor for both low bone mass and fractures in diabetic and non-diabetic subjects. Considering that PA is the most ubiquitous FA in humans (including their bone marrow), and since PA induces autophagy and/or apoptosis in other systems (Choi et al., 2009; Martino et al., 2012; Xie et al., 2012), in this study we tested whether it also induces these processes in Ob. Here we used a dose of PA that corresponds to the concentration secreted by adipocytes into the media (Elbaz et al., 2010), was already used in previous studies looking at PA-induced lipotoxicity in bone cells (Elbaz et al., 2010; Kim et al., 2008), and closely corresponds to the levels of PA found in bone marrow of human subjects (Griffith et al., 2009). We found that PA induces autophagy and apoptosis in a time- and dose-dependent manner and also demonstrated that these two phenomena could be prevented by directly targeting key signaling pathways.

In other cell models, autophagy is a survival mechanism that removes damaged organelles during cell stress and nutrient deprivation (Levine and Klionsky, 2004). Autophagy can also be a defense mechanism against apoptosis by recycling nutrients, maintaining cellular energy homeostasis, degrading toxic cytoplasmic constituents, and removing intracellular pathogens (Levine and Klionsky, 2004). In bone cells, autophagy has been recently proposed as a key process in skeletal maintenance (Hocking et al., 2012), however studies looking at the role of autophagy in bone cells remain scarce.

Contrary to autophagy, apoptosis in bone cells has been well studied and is a key process in both osteoblasts and osteoclasts. All osteoclasts ultimately undergo apoptosis mediated by well-defined pathways (Fong et al., 2013; Tanaka et al., 2006). In contrast, osteoblast apoptosis increases during aging, after corticosteroid treatment and in osteoporotic bones; however, the underlying mechanisms are not only poorly understood but also difficult to quantify (Jilka et al., 2007).

Recently, it has been proposed that the progressive accumulation of adipocytes and their secreted factors within the bone marrow milieu could exert a toxic effect on other cells in their vicinity (Payne et al., 2007). PA can induce apoptosis in Ob *in vitro*, a process that can be prevented by adding an inhibitor of FA synthase to the media (Elbaz et al., 2010). Interestingly, PA also activates autophagy in several cell models such as beta cells (Martino et al., 2012) and endothelial cells (Khan et al., 2012). In these two cell types, PA led to a significant dose- and time-dependent decline in cell function and survival.

There is only one report of the apoptotic pathways activated by PA in Ob (Kim et al., 2008). The authors reported that PA induces apoptosis in Ob due to impaired activation of ERK and that this process could be prevented through activation of AMP-activated kinase (AMPK). This evidence contradicts the usual concept that osteoblast apoptosis is predominantly mitochondrial (Duque et al., 2004; Manolagas, 2000). Therefore, to identify the apoptotic pathway activated by PA in our cell model, we used a protein array approach to identify the apoptotic pathways activated by PA. PA activated a Fas-related apoptotic pathway in Ob, which is also activated in serum-deprived Ob (Duque et al., 2004) and in Ob exposed to corticosteroids (Rippo et al., 2010). In PA-mediated apoptosis, we also identified increased levels of three of the pro-apoptotic proteins of mitochondrial origin and an increase in the Bax/Bcl2 ratio, which indicate higher susceptibility to apoptosis. In fact, this higher susceptibility to apoptosis could also be associated with changes in the level of cardiolipin, which constitutes 20% of the inner mitochondrial membrane and is known to release cytochrome C during palmitate-induced apoptosis in other cell models (Buratta et al., 2008). Although cardiolipin has not been previously associated with palmitate-induced apoptosis in bone cells, the fact that high levels of cytochrome C are observed in the cytoplasm of palmitate-treated Ob could constitute an indirect marker of changes in cardiolipin levels. This possibility, together with the elucidation of the role of cardiolipin in apoptosis in bone cells, will be a subject of future studies.

In terms of the molecular mechanisms of PA-induced apoptosis, several studies have revealed a role for JNK pathway in the pathogenesis of PA-induced apoptosis in endothelial cells (Xie et al., 2012), pancreatic β cells (Xiang et al., 2012) and hepatocytes (Luo et al., 2012). For instance, in hepatocytes PA causes apoptosis by activating the proapoptotic protein Bax in a JNK-dependent manner (Malhi and Gores, 2008). After identifying higher levels of phosphorylated JNK in PA-treated Ob, we attempted to block this effect using an inhibitor of JNK phosphorylation, SP600125, which has previously been used to inhibit JNK-induced lipoapoptosis in other cell models (Luo et al., 2012). The protection from apoptosis obtained by blocking the JNK pathway suggests a new potential therapeutic target to prevent fat-induced apoptosis in Ob.

We also sought to characterize the role of autophagy in PA-induced lipotoxicity in Ob. High levels of autophagy were observed in Ob shortly after exposure to PA, which we were able to inhibit using the PI3K inhibitor 3MA. Autophagy activation depends on beclin and class III PI3K (He and Levine, 2010), and therefore PI3K is a critical target for autophagy inhibition. 3MA blocks PI3K and interferes with two different steps in the autophagic/lysosomal pathway: inhibition of autophagosome formation and inhibition of the lysosomal breakdown of macromolecules (Caro et al., 1988). Our EM and fluorescence data showed that 3MA blocked PA-induced autophagosome formation concomitantly with a reduction in the expression of Cytochrome C in the cytosol, thus identifying another potential therapeutic target to protect Ob from lipotoxicity and cell death.

In summary, our *in vitro* data suggest that PA induces both autophagy and apoptosis in Ob. Our findings point to a new therapeutic approach since understanding the molecular mechanisms of lipotoxicity in bone would enable us to identify new targets to prevent autophagy and apoptosis, which are induced by the presence of FA in the bone marrow milieu and are associated with the cellular changes observed in aging and osteoporosis.

## Materials and Methods

### Normal human osteoblasts (Ob)

Ob and media were purchased from Lonza (CC-2538, Basel, Switzerland). Cells at passages between three and six from time of marrow harvest were used in these experiments. Ob were plated in growth media at 37°C in a humidified atmosphere of 5% CO_2_. Growth media was composed of osteoblast basal medium media (C-3208, Lonza) containing 10% of FBS, 0.1% ascorbic acid, and 0.1% gentamicin. The cells were allowed to grow for 2 days after which media was changed to a combination of osteoblast basal medium and a growth medium kit (CC-4193, Lonza) containing hydrocortisone (200 µM) and 5 ml β-glycerophosphate (1 M).

### Ob treatment

Ob were plated at a density of 4×10^5^ cells/cm^2^ in six-well plates containing osteoblast growth media at 37°C in a humidified atmosphere of 5% CO_2_. After reaching 80% confluence in basal media, media was replaced with osteoblast growth media with or without PA (100, 250, and 500 µM). PA stock solution was prepared and treatment was performed as previously described (Elbaz et al., 2010; Kim et al., 2008). Briefly, PA was dissolved in ethanol at 37°C for the stock solution (10 mM) and then dissolved in phosphate buffered saline (PBS) containing 175 mg/ml (2.5 mM) FA-free bovine serum albumin (BSA) to obtain a 10 µM FA stock solution. The molar ratio of FA to BSA was 4:1. Control cells were also treated with BSA. To prevent autophagy, cells were treated with 3-methyladenine (3MA) (5 mM). To prevent apoptosis, cells were treated with SP600125 (10 µM).

### Effect of PA on cell survival

Ob were plated in 96-well plates at a density of 1×10^4^ cells per well. After an initial 24 h period in growth media (time 0), cells were treated with either PA (100, 250 and 500 µM) or with vehicle alone. Cell survival was evaluated at timed intervals (24, 48 and 72 h) using an MTS tetrazolium assay (Promega, Madison, WI, USA) according to the manufacturer's instructions. Survival was calculated as percentage change compared with control.

### Identification of apoptosis

Terminal deoxynucleotidyl transferase dUTP nick end labeling (TUNEL) assay was performed in cells treated with either PA or vehicle alone for 48 and 72 h. The cells were harvested by centrifugation and TUNEL staining was performed according to the manufacturer's instructions (Roche, Basel, Switzerland). Briefly, cells were fixed with 4% paraformaldehyde for 20 min at room temperature, followed by permeabilization buffer (triton X-100) at 37°C for 30 min. Samples underwent reaction with terminal deoxynucleotide transferase (TdT) in the presence of fluorescein-conjugated nucleotide substrate for 1 hour at 37°C. Level of fluorescence was determined using a BD FACS Caliber flow cytometer (BD Biosciences, CA, USA) with Cell Quest software.

### Apoptosis protein array

Cells were treated with either PA (100, 250 and 500 µM) or vehicle for 48 h. Total protein was extracted from the cells and 300 mg of protein were analyzed using a protein array with antibodies against 43 apoptosis-related proteins (Human Apoptosis Array Kit, RayBiotech Inc, USA). After incubation with a cocktail of biotinylated antibodies and labeled-streptavidin, the signal was detected by chemiluminescence using an Axon GenePix AGP 4000B. Spot signal intensity was analyzed using the GenePix Pro 6.0 software and the RayBio® Antibody Array Analysis Tool. Intensities among different arrays were normalized using an internal control as suggested by manufacturer. Intensity values above the average intensities of the negative controls were taken as positive signal and signal intensities greater than 2-fold or higher than untreated cells were considered to be a significant change. This experiment was repeated two times. Results were reported as mean ± SEM for at least three analyses for each sample.

### Western blotting

After all different treatment conditions, Ob were lysed using lysis buffer and protease inhibitor tablets (Roche Diagnostics, Indiana, USA) and centrifuged at 13,000 *g* for 10 min to remove insoluble material. Before electrophoresis, protein concentrations were measured using BCA kit (Thermo Scientific Pierce, USA) and then dissolved in SDS electrophoresis buffer (Bio-Rad, Hercules, CA). Ten micrograms of protein per well were separated on SDS-polyacrylamide gels and subsequently electro transferred to a PVDF membrane (Amersham, UK) for 50 min at 300 mA. After blocking with PBS containing 0.1% Tween 20 and 5% BSA, membranes were incubated overnight at 4°C using primary antibodies against LC3-I and II and Tubulin (Santa Cruz, CA, USA) for autophagy and antibodies against Bax, cytoplasmic cytochrome C, phosphorylated c-Jun-N terminal kinase (p-JNK), B-cell lymphoma 2 (Bcl2) (Cell Signaling Technology, Arundel, Queensland) for apoptosis, with glyceraldehyde 3-phosphate dehydrogenase (GAPDH) as the loading control. The bound antibodies were detected with the corresponding secondary antibodies (1:10,000) conjugated with horseradish peroxidase. Blots were developed by enhanced chemiluminescence (Super-Signal West Pico Chemiluminescent Substrate, Thermo Scientific, USA). Quantification was performed using Image J Software. Relative intensities were determined after correcting the protein of interest with either GAPDH. Experiments were repeated three times.

### Confocal microscopy

To identify cytochrome C expression, Ob were grown and treated exactly as above for LC3 staining. A primary antibody against mitochondrial cytochrome C (Santa Cruz Biotechnology, Santa Cruz CA, USA) was used, together with a secondary Alexa 555 conjugated donkey anti-mouse antibody (Invitrogen, Victoria, Australia). Control samples were incubated with secondary antibody alone. Samples were analyzed using Leica SP5 confocal microscope. Intensity was calculated using image J software.

The number of LC3-positive puncta/cell was used as a measure of autophagy (Tanida et al., 2004). Cells were grown on coverslips in the presence or absence of PA (100, 250 and 500 µM) for 24–72 h. Cells were then fixed in 4% paraformaldehyde for 20 min and washed three times with PBS, permeabilized with 0.1% triton X-100 for 10 min, washed and blocked with 5% BSA for 1 h. A primary anti-LC3 antibody was used, together with a secondary Alexa 488 conjugated donkey anti-rabbit antibody (Invitrogen, Victoria, Australia). Control samples were incubated with secondary antibody alone. Samples were analyzed using a Leica SP5 confocal microscope. For quantification of LC3-positive puncta, numbers were determined by counting ten fields from ten different cells in two independent coverslips in each condition. This experiment was repeated three times.

### Electron microscopy (EM)

Cells were treated with PA (100, 250 and 500 µM) and analyzed by EM at 24, 48 and 72 h. Ob were grown on coverslips and fixed in Karnovsky's fixative (2.5% glutaraldehyde and 2.4% formaldehyde [freshly prepared from paraformaldehyde] in 0.1 M MOPS buffer pH 7.4). Cells were post-fixed in 2% buffered osmium tetroxide, dehydrated in a graded series of ethanols and finally embedded in TAAB TLV epoxy resin, which was polymerized at 70°C for 10 hours. Ultrathin sections (70 nm) were cut using a Leica Ultracut E or Ultracut UC6rt ultra microtome, collected on 300-mesh copper grids and stained with 2% uranyl acetate in 50% ethanol, followed by Reynold's lead citrate. Sections were examined in a Philips CM 120 BioTWIN transmission electron microscope at 100 kV. Images were recorded with a SIS Morada digital camera using iTEM software. To evaluate autophagosome formation cells were grown on coverslips treated with PA (100, 250 and 500 µM) with or without 3MA for 48 h. The samples were grown on coverslips and were fixed in Karnovsky's fixative, processed and imaged as before. For quantitation of autophagosomes, the data obtained from a minimum of 20 independent cross-sectional cells was averaged (mean ± SD).

### Statistical analysis

All data are expressed as mean ± SD of three replicate determinations. Unless otherwise stated, all experiments were repeated three times. Statistical analysis was performed by one-way ANOVA or Student's t-test. A probability value of *P*<0.05 was considered statistically significant.
